# Disease-Specific α-Synuclein Seeding in Lewy Body Disease and Multiple System Atrophy Are Preserved in Formaldehyde-Fixed Paraffin-Embedded Human Brain

**DOI:** 10.3390/biom13060936

**Published:** 2023-06-02

**Authors:** Ain Kim, Ivan Martinez-Valbuena, Jun Li, Anthony E. Lang, Gabor G. Kovacs

**Affiliations:** 1 Tanz Centre for Research in Neurodegenerative Diseases, University of Toronto, Toronto, ON M5T 0S8, Canada; ain.kim@mail.utoronto.ca (A.K.); ivan.martinez@utoronto.ca (I.M.-V.); junjl.li@utoronto.ca (J.L.); anthony.lang@uhnresearch.ca (A.E.L.); 2 Department of Laboratory Medicine and Pathobiology, University of Toronto, Toronto, ON M5S 1A8, Canada; 3 Krembil Brain Institute, University Health Network, Toronto, ON M5T 0S8, Canada; 4 Edmond J. Safra Program in Parkinson’s Disease and the Morton and Gloria Shulman Movement Disorders Clinic, Toronto Western Hospital, Toronto, ON M5T 2S6, Canada; 5 Laboratory Medicine Program, University Health Network, Toronto, ON M5G 2C4, Canada

**Keywords:** alpha-synuclein, protein extraction, FFPE, SAA, seeding behaviour

## Abstract

Recent studies have been able to detect α-synuclein (αSyn) seeding in formaldehyde-fixed paraffin-embedded (FFPE) tissues from patients with synucleinopathies using seed amplification assays (SAAs), but with relatively low sensitivity due to limited protein extraction efficiency. With the aim of introducing an alternative option to frozen tissues, we developed a streamlined protein extraction protocol for evaluating disease-specific seeding in FFPE human brain. We evaluated the protein extraction efficiency of different tissue preparations, deparaffinizations, and protein extraction buffers using formaldehyde-fixed and FFPE tissue of a single Lewy body disease (LBD) subject. Alternatively, we incorporated heat-induced antigen retrieval and dissociation using a commercially available kit. Our novel protein extraction protocol has been optimized to work with 10 sections of 4.5-µm-thickness or 2-mm-diameter micro-punch of FFPE tissue that can be used to seed SAAs. We demonstrated that extracted proteins from FFPE still preserve seeding potential and further show disease-specific seeding in LBD and multiple system atrophy. To the best of our knowledge, our study is the first to recapitulate disease-specific αSyn seeding behaviour in FFPE human brain. Our findings open new perspectives in re-evaluating archived human brain tissue, extending the disease-specific seeding assays to larger cohorts to facilitate molecular subtyping of synucleinopathies.

## 1. Introduction

Synucleinopathies are neurodegenerative disorders that are characterized by the loss of neurons and deposition of misfolded α-synuclein (αSyn) in various cell types. αSyn is a 140-amino acid cytoplasmic protein encoded by the *SNCA* gene and is localized in presynaptic terminals of neurons [1]. Its pathogenic form is composed of insoluble β-sheet-rich structures and phosphorylation at the Ser129 residue [2,3]. Synucleinopathies comprise Parkinson’s Disease (PD), Parkinson’s Disease with dementia (PDD), and dementia with Lewy body (DLB), together grouped as Lewy body diseases (LBDs) and multiple system atrophy (MSA). Neuropathologically, LBDs are characterized by inclusion body formation in neurons, specifically in Lewy bodies (LB) and Lewy neurites [4,5], collectively referred to as Lewy pathology. Clinical symptoms of LBDs include bradykinesia, rigidity, resting tremor, postural instability, and other non-motor symptoms. On the other hand, MSA is neuropathologically characterized by the accumulation of αSyn in oligodendrocytes, termed glial cytoplasmic inclusions (GCI) [6]. Clinically, patients with MSA have a more rapid progression of disease, with motor and cerebellar symptoms and autonomic failure [7].

This diverse nature of synucleinopathies stems from the same αSyn protein, but recent discoveries on the structural and biochemical differences have led to the hypothesis that distinct conformations of misfolded αSyn result in different polymorphs or strains [8,9,10,11]. Distinct polymorphs have been shown to exhibit different rates of cell-to-cell propagation, postulated to take part in determining the clinical and neuropathological phenotypes in synucleinopathies, as occurs in prion disorders [12,13]. Based on this, the seeding behaviour of misfolded αSyn in LBD and MSA has been recapitulated in vitro using seed amplification assays (SAAs), such as real-time quaking-induced conversion (RT-QuIC) or protein misfolding cyclic amplification (PMCA). SAAs are shaking-based cyclic amplification techniques that exploit the property of self-propagation, amplifying αSyn seeds that can be detected and characterized with high sensitivity and specificity in post-mortem brain tissue and in vivo samples like cerebrospinal fluid and skin [14,15]. SAAs allow real-time detection of aggregated αSyn using thioflavin T (ThT) that binds to β-sheet structures of misfolded αSyn. ThT fluorescence is measured at multiple time points, allowing the analysis of kinetic differences between different subjects [16]. Different temperatures, pH, salt, and buffer composition can alter the in vitro fibril growth and sensitivity of the assay, further enabling selective detection of pathogenic seeds [17,18,19]. Recent studies from our group have identified optimal assay conditions that favour either LBD or MSA seeding activity and demonstrated that not only does seeding differ between synucleinopathies but also between regions within subjects [20,21].

Despite the great sensitivity and specificity shown by these assays, post-mortem frozen brain tissues are less available with the widespread decrease in autopsy rates and the rising cost of storage of these specimens in ultra-low temperature freezers. To overcome this limitation, FFPE tissues can be utilized in seeding assays to allow further evaluation of large cohorts. FFPE remains the gold standard for tissue preservation that is widely used in hospitals, brain banks, and research laboratories, allowing neuropathological evaluations of the human post-mortem brain. However, the formaldehyde-fixed tissues have various crosslinks between macromolecules and, most notably, Schiff base formation between proteins and formaldehyde [22]. These inter- and intramolecular crosslinks improve the stability of proteins which is crucial for tissue preservation. Although FFPE tissues retain biological information that can allow an understanding of underlying disease mechanisms, they are limited to histological analysis once tissues are fixed. Despite this limitation, several studies have reported the successful extraction and recovery of disease-associated prion protein (PrP) from FFPE or formaldehyde-fixed tissues [23,24]. Because the immuno-detection of the misfolded PrP may be difficult in archival tissues, some groups have used FFPE tissues to sensitively detect misfolded PrP in chronic wasting disease (CWD) and sporadic Creutzfeldt–Jakob disease (sCJD) using SAAs [25,26]. Although formaldehyde-fixation decreases protein extraction efficiency, extraction of misfolded PrP from FFPE tissue was sufficient using a simple PBS buffer conventionally used to homogenize frozen tissue and subsequent SAAs were not limited by the low extraction efficiency, given the high infectivity profile of misfolded prion proteins [27].

In addition, a handful of studies have successfully detected and amplified misfolded αSyn in FFPE skin, submandibular glands, and the gastrointestinal tract [28,29,30]. However, the authors consistently reported limitations in the extraction efficiency of proteins, presumably due to formaldehyde crosslinks formed between proteins and other macromolecules during fixation [31,32,33], remnant paraffin residues in tissue, or the low amount of tissue used (5–6-µm tissue sections). Different techniques have been reported to remove paraffin residues and reverse formaldehyde crosslinks using various protein extraction buffers [34,35,36,37,38]. Among the different techniques, the application of thermal energy via antigen retrieval and extraction buffers corrected for ionic strength differences were determined to be the key elements for efficient protein extraction [39,40,41,42].

Considering that FFPE tissues can provide the advantage of linking seeding behaviour with neuropathologically characterized brain regions and allow the investigation of larger cohorts, we aimed to establish a streamlined protein extraction protocol specific for the evaluation of αSyn seeding in FFPE human brain tissue.

## 2. Materials and Methods

### 2.1. Case Selection

Six subjects with LBD (4 females), 2 subjects with MSA (1 female), and 1 subject lacking pathology (1 female) were selected from the University Health Network-Neurodegenerative Brain Collection (UHN-NBC, Toronto, ON, Canada). The temporal cortex of 1 LBD subject was used for optimization, and the substantia nigra was used for validation. For the rest of the experiments, the temporal cortex of LBD subjects and cerebellum of MSA subjects were used in this study, in addition to the parietal cortex and cerebellum white matter of a control subject. Case selection was based on a systematic neuropathological evaluation using the diagnostic criteria of neurodegenerative conditions and co-pathologies [4,43]. Demographics and neuropathological evaluations are summarized in Table 1. Human brain tissues were collected during autopsy with informed consent from patients or their relatives and approved by institutional review boards. All tissues were fixed in formaldehyde for 2 weeks and embedded in paraffin for staining and storage for research purposes. This study was approved by the University Health Network Research Ethics Board (Nr. 20-5258).

### 2.2. Immunohistochemistry

FFPE tissues were cut into 4.5-µm-thick sections using a microtome and immuno-stained with the monoclonal 5G4 anti-mouse antibody [44], which labels only the disease-associated αSyn (1:4000; 5 min pre-treatment with 80% formic acid; Roboscreen, Leipzig, Germany). Target retrieval was performed using the DAKO EnVision FLEX Target Retrieval Solution. The DAKO EnVision detection kit, EnVision FLEX peroxidase-blocking solution, 3,3′-Diaminobenzidine chromogen, and EnVision FLEX+ mouse linker (Dako, Glostrup, Denmark) was used to visualize the antibody reactions.

Digital images were obtained with TissueScope^TM^ LE120 and TissueSnap^TM^ (Huron, Saint Jacobs, ON, Canada) and cropped using HuronViewer software (Figure S1).

### 2.3. Sample Collection

To test the protein extraction efficiency, FFPE tissues were prepared by (1) cutting 4.5-µm-thick sections on positively charged glass slides totalling up to 45-µm of tissue, (2) cutting 4.5-µm-thick scrolls in 1.5 mL low protein binding tubes (Sarstedt, Nümbrecht, Germany) totalling up to 45-µm of tissue, (3) collecting 4-mm micro-punch of the FFPE block, (4) collecting 2-mm micro-punch of the FFPE block, and (5) collecting 4-mm micro-punch of the formaldehyde-fixed tissue. To ensure that FFPE blocks were re-usable after micro-puncture, paraffin was melted prior to micro-punch collection and the rest of the tissue was re-embedded. To melt the wax, FFPE blocks were placed on Tissue-Tek^®^ base moulds and incubated in the paraffin-melting chamber of the embedding machine (Sakura Finetek, Nagano, Japan) for 10 min at 62 °C. A disposable biopsy punch with a plunger (Integra Miltex, York, PA, USA), conventionally used for skin biopsies, was used to collect micro-punches of the FFPE tissue block. Biopsy punches were safely discarded after collecting each sample to avoid cross-contamination.

### 2.4. Deparaffinization

To test the efficiency of paraffin removal, two methods were tested: (1) xylene-ethanol and (2) heptane-methanol. For xylene-ethanol, tissues were incubated twice in fresh xylene for 10 min at room temperature, then rehydrated in a series of graded ethanol (90%, 80%, 70%, 50%) and in distilled water at room temperature for 5 min each. Tissue on slides was placed in racks and submerged into the staining dish, while for tissue in low protein binding tubes (C-tubes (Miltenyi BioTec, Auburn, CA, USA) in the optimized protocol), the supernatant was carefully removed and replaced with the next solution. For heptane-methanol, tissues were incubated in heptane for 10 min at room temperature, vortexed for 10 s in 5-min intervals, then mixed with 5% *v*/*v* methanol and vortexed for 10 s. The supernatant was then removed, and tissues were dried for 5 min at room temperature in a biosafety cabinet. Tissue on slides was placed in racks and dipped into the staining dish several times.

After deparaffinization and rehydration, tissue on slides was carefully scraped with a microtome blade coated in Sigmacote (Sigma, St. Louis, MI, USA) for 2 min and dried for 20 min in a biosafety cabinet. Scraped tissues were transferred to a clean 1.5 mL low protein binding tube and weighed.

### 2.5. Antigen Retrieval and Dissociation

The FFPE Tissue Dissociation Kit (Miltenyi BioTec, Auburn, CA, USA) was used for antigen retrieval and dissociation according to the manufacturer’s instructions with slight modifications. In brief, deparaffinized tissues were transferred to C-tubes and, with the provided buffer, incubated in an 80 °C water bath for 75 min. Once the samples were cooled, the supernatant was carefully removed, and with the provided buffer and enzymes, the samples were dissociated on the gentleMACS™ Dissociator (Miltenyi BioTec, Auburn, CA, USA). The dissociated tissues were then transferred into 1.5 mL low protein binding tubes and centrifuged at 4 °C for 5 min at 5000× *g*. The supernatant was discarded, and the tissue pellet was resuspended in PBS. The tubes were centrifuged once more, at 4 °C for 5 min at 5000× *g*, and the supernatant was removed. The resulting tissue pellet was weighed and used for protein extraction.

### 2.6. Protein Extraction and Quantification

To test the efficiency of different protein extraction buffers, deparaffinized tissues were weighed and carefully transferred to Precellys^®^ 2 mL Protein Safe Soft Tissue Homogenizing tubes containing zirconium oxide beads (CK14; Bertin Corp, Rockville, MD, USA). Ice-cold 10% weight/volume (*w*/*v*) extraction buffers (Table 2) were added into the tube and placed in the Minilys bead homogenizer (Bertin Technologies SAS, Montigny-le-Bretonneux, France) for 30 s at high speed. The tubes were put on ice for 5 min, and the homogenization was repeated 2 more times. For tissues processed with antigen retrieval and dissociation, the tissue pellet was weighed and resuspended in ice-cold 10% *w*/*v* PBS before being transferred to the homogenizing tubes. Following homogenization, the tissue homogenate was transferred to 1.5 mL low protein binding tubes, incubated for 10 min on ice, and centrifuged for 10 min at 4 °C 10,000× *g* as previously described [20]. The supernatant was collected in a single 1.5 mL low protein binding tube and then aliquoted in 0.5 mL low protein binding tubes to avoid excessive freeze-thaw cycles.

Following protein extraction, bicinchoninic acid protein (BCA) assay (Thermo Scientific, Waltham, MA, USA) was performed to determine the concentration of total protein in each sample.

### 2.7. αSyn SAA Protocol

SAA was performed as previously described [20]. In brief, the SAA reaction mixture was composed of 40 mM phosphate buffer (pH 8.0), 350 mM Na_3_Citrate (Sigma, St. Louis, MI, USA), 0.1 mg/mL K23Q human recombinant αSyn (Impact Biologicals, PA, USA) and 10 µM ThT (Sigma, St. Louis, MI, USA). For this study, K23Q human recombinant αSyn was used, as it has been shown to be less prone to spontaneous aggregation in the absence of seed [45]. Recombinant αSyn was thawed from −80 °C storage and filtered through a 100 kDa Amicon ultra-0.5 centrifugal filter unit (Sigma, St. Louis, MI, USA). For serial dilution experiments, 1.25, 2.5, 5 and 10 µg of total protein were used as seeds for the SAA reaction. For testing the transmissibility of αSyn seeding differences by templating, 2 µL of SAA end-product were used as seeds for subsequent SAA reaction. For every plate, a positive control (1 µg of pre-formed αSyn fibrils) and a negative control (deionized water) were included. The plate was sealed with DNase-/RNase-free clear polyolefin sealing tape (Thermo Scientific, Waltham, MA, USA).

To evaluate the differential seeding behaviour between LBD and MSA subjects, two SAA protocols were performed [20,21]. In the first protocol, an LBD-favouring SAA, the plate was incubated at 42 °C with cycles of 1 min shaking and 1 min rest. In the second protocol, an MSA-favouring SAA, the plate was incubated at 37 °C in a BMG FLUOstar Omega plate reader with cycles of 1 min shaking (400 rpm double orbital) and 14 min rest. In both protocols, ThT fluorescence measurements (450 ± 10 nm excitation and 480 ± 10 nm emission, bottom read) were taken every 15 min for a period of 90 h.

### 2.8. Transmission Electron Microscopy

Ten microliters of αSyn SAA end-products were loaded onto freshly glow-discharged 400 mesh carbon-coated copper grids (Electron Microscopy Sciences, Hatfield, PA, USA) and adsorbed for 1 min. Once dry, the grids were visualized using a Talos L120C transmission electron microscope (Thermo Scientific, Waltham, MA, USA) using an acceleration voltage of 200 kV. Electron micrographs were recorded using an Eagle 4kx4k CETA CMOS camera (Thermo Scientific, Waltham, MA, USA).

### 2.9. Statistical Analysis

All statistical analyses were performed using Prism (v.9, GraphPad Software, San Diego, CA, USA). Kinetic curves were plotted, and the area under the curve (AUC) was obtained using Prism. Kinetic curves were compared using unpaired two-tailed parametric *t*-test or one-way ANOVA with Tukey’s multiple comparison test. Significance was declared at *p* < 0.05.

## 3. Results

**Figure 1:**
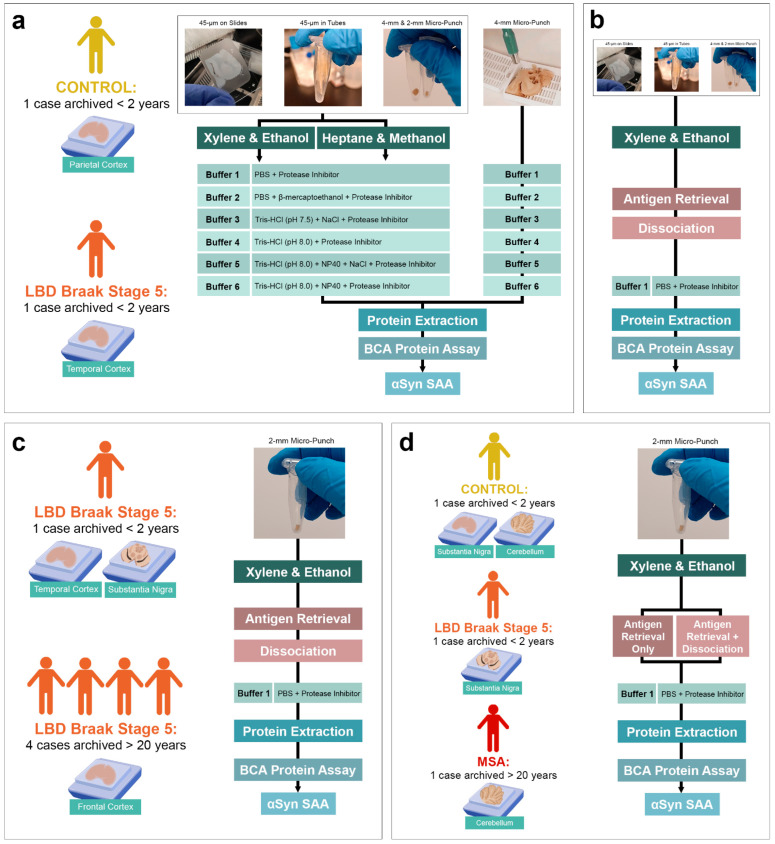
Optimization Schematic of FFPE Protein Extraction. (**a**) Testing protein extraction efficiency using FFPE and formaldehyde-fixed temporal cortex of test LBD subject with three different tissue preparations, two different deparaffinization and rehydration methods, and six different extraction buffers compared to a control subject (both archived for <2 years); (**b**) Introduction of heat-induced antigen retrieval and dissociation to increase protein yield extracted with simple PBS buffer; (**c**) Application of optimized extraction protocol to evaluate protein extraction efficiency and αSyn seeding in different regions and LBD subjects(archived > 20 years); (**d**) Evaluation of αSyn disease-specificity in the substantia nigra of LBD (archived < 2 years), the cerebellum of MSA (archived > 20 years) and the parietal cortex and cerebellum of control (archived < 2 years) subjects and comparison of seeding between tissues processed with antigen retrieval-only or both antigen retrieval and dissociation.

To evaluate whether αSyn seeding is preserved in FFPE human brain tissue, we tested different tissue preparations, deparaffinization methods and extraction buffers that have been previously reported (Figure 1a). However, we observed low or inconsistent seeding activity with these methods. To determine the most efficient protein extraction method that yields the highest protein concentration while preserving the disease-specific αSyn seeding behaviour, we then tested a novel approach using a heat-induced antigen retrieval and dissociation process using a commercially available kit (Figure 1b). Using this novel approach, we observed an improvement in the protein extraction efficiency compared to conventional extraction protocols and an increase in αSyn seeding. We validated our optimized protocol using FFPE brain tissue from five LBD (four archived > 20 years) and two MSA subjects (one archived > 20 years) (Figure 1c,d).

### 3.1. Protein Extraction and SAA from FFPE Human Brain Tissue

#### 3.1.1. Comparison of Protein Extraction Efficiency Using Different Tissue Preparation, Deparaffinization and Extraction Buffers

As previous FFPE protein extraction protocols have used 5- or 6-µm-thick FFPE sections either mounted on glass slides or scrolls in tubes, we tested the protein extraction efficiency using a total of ten 4.5-µm-thick FFPE temporal cortex sections deparaffinized on positively charged glass slides or directly placed into low protein binding tubes. Alternatively, to conserve the FFPE tissues as much as possible while maximizing the amount of tissue used, we also tested the protein extraction efficiency from a 4-mm micro-punched FFPE tissue. With slight modifications to previously reported protein extraction buffers [37], we compared the protein extraction efficiency of six different extraction buffers made in-house. The composition of different buffers is outlined in Table 2.

**Figure 2:**
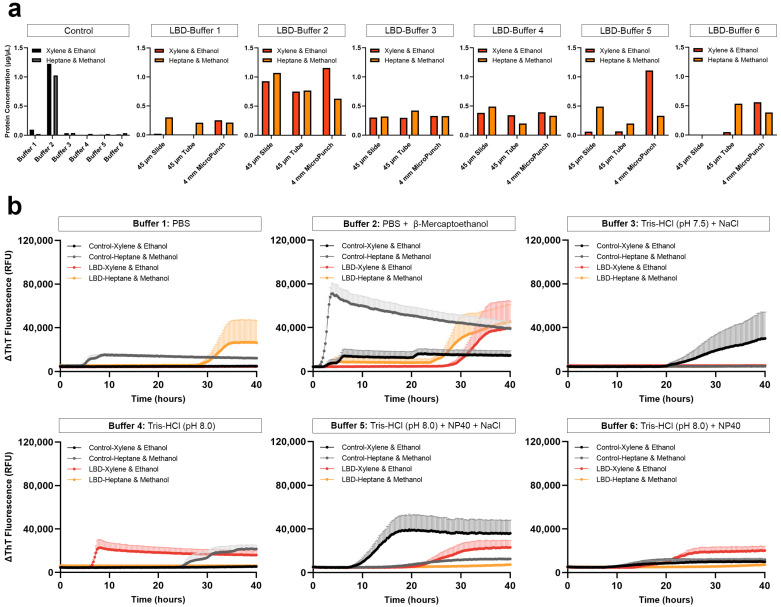
Comparison of FFPE protein extraction efficiency and subsequent αSyn seeding using various tissue preparations, deparaffinization, and extraction methods. (**a**) Protein yield obtained with either xylene-ethanol or heptane-methanol deparaffinization, extracted from 4-mm micro-punch of the parietal cortex of test control subject and 45-µm FFPE sections on slides, in tubes, or 4-mm FFPE micro-punch of the temporal cortex of test LBD subject, using six different extraction buffers; (**b**) Subsequent αSyn seeding potential of the extracted proteins from 4-mm FFPE micro-punch of LBD temporal cortex and control parietal cortex.

Using the xylene-ethanol deparaffinization, we found that protein extraction from 4-mm micro-punch of the control tissue with buffer 2 yielded the highest amount of protein, lower yield with buffer 1 and the lowest with buffers 3, 4, 5 and 6. In LBD tissue, proteins extracted with buffer 1 yielded the lowest amount of protein across all tissue preparation types and at the other extreme, BCA showed the highest protein measurement with buffer 2 (Figure 2a). It is important to note that although 0.01% β-mercaptoethanol is compatible with BCA, it is possible that the reducing agent may have distorted total protein measurement, falsely making the values higher. When buffer 1, 2, 5 or 6 was used, the protein concentrations of FFPE micro-punch were higher than those from FFPE sections. On the other hand, the FFPE micro-punch had a similar protein concentration to those from FFPE sections when buffers 3 or 4 were used with xylene-ethanol deparaffinization. Between FFPE sections on slides and in tubes, protein concentrations had subtle differences across the extraction buffers, except buffer 2, where FFPE sections deparaffinized on slides had higher concentrations than those in tubes.

Based on previous studies highlighting the importance of complete paraffin removal without using toxic solvents like xylene that can impede protein extraction [46], we tested the protein extraction efficiency using an alternative deparaffinization and rehydration method consisting of heptane-methanol. For control tissues, all extraction buffers, except buffers 1 and 2, yielded higher protein concentrations when deparaffinized with heptane-methanol compared to those deparaffinized with xylene-methanol. For LBD tissues, all extraction buffers, except buffer 4, yielded higher protein concentrations in FFPE sections when deparaffinized with heptane-methanol compared to those deparaffinized with xylene-ethanol. When buffer 4 was used with heptane-methanol deparaffinization, FFPE sections on slides had higher protein concentrations, while FFPE sections in tubes had lower concentrations compared to those deparaffinized with xylene-ethanol. For proteins extracted from 4-mm FFPE micro-punch, concentrations were lower in all extraction buffers with heptane-methanol deparaffinization, except buffer 3, compared to those deparaffinized with xylene-ethanol. When buffer 3 was used, protein concentrations were similar to those deparaffinized with xylene-ethanol or heptane-methanol. However, with heptane-methanol deparaffinization, we observed a thick layer of paraffin residue on top of the buffers at the end of the protein extraction, regardless of the yield of protein extracted.

#### 3.1.2. Seeding of αSyn Extracted Using Different Tissue Preparation, Deparaffinization and Extraction Buffers

The heptane-methanol deparaffinization yielded varying protein concentrations across all extraction buffers, but we observed consistent improvement in extraction using 4-mm FFPE micro-punch across all extraction buffers using xylene-ethanol deparaffinization. Therefore, we used the 4-mm FFPE micro-punch of the test LBD subject and control subject to compare the seeding of αSyn extracted using different deparaffinization and extraction buffers. With heptane-methanol deparaffinization, controls were positive when extracted with buffers 1, 2, or 4 while αSyn from LBD tissues showed seeding at around 30 h with buffer 1, inconsistent seeding at around 28 h with buffer 2, and no seeding with buffers 3, 4, 5, or 6 (Figure 2b). We suspect that since seeding requires a bona fide liquid-air interface, insufficient removal of paraffin using the heptane-methanol deparaffinization may have led to the formation of a thin paraffin layer in the reaction mixture, resulting in the lack of seeding. For tissues deparaffinized with xylene-ethanol, controls were positive when extracted with buffers 2, 3, 5 or 6 while misfolded αSyn extracted from LBD tissue with buffer 1 showed negative seeding and positive seeding at 30 h with extraction buffer 2. Tris-containing extraction buffers 4, 5 and 6, but not buffer 3, showed αSyn seeding. When proteins were extracted with only Tris-HCl at pH 8.0 (buffer 4), we observed αSyn seeding activity at 5 h, while those extracted with NaCl and NP40 (buffer 5) seeded at 20 h and those extracted with NP40 (buffer 6) seeded at 12 h. Altogether, we observed low or delayed seeding activity in tissues deparaffinized with xylene-ethanol but mostly negative αSyn seeding in tissues deparaffinized with heptane-methanol with visibly incomplete deparaffinization. Therefore, we resorted to using xylene-ethanol henceforward.

### 3.2. Protein Extraction and SAA from Formaldehyde-Fixed Human Brain Tissue

**Figure 3:**
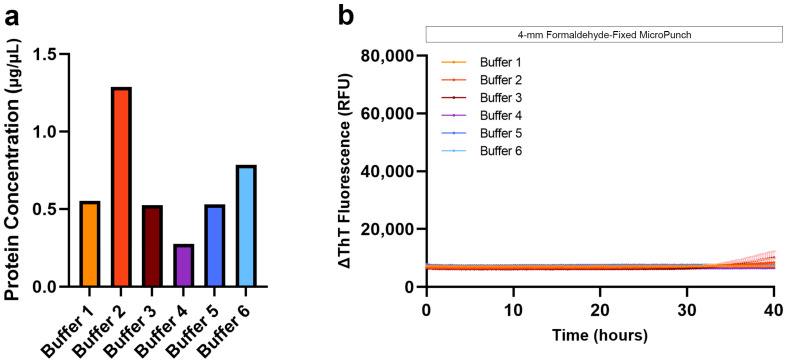
Protein extraction efficiency of different extraction buffers using formaldehyde-fixed human brain tissue and subsequent αSyn seeding potential. (**a**) Protein yield obtained from 4-mm micro-punch of formaldehyde-fixed FFPE temporal cortex of LBD, extracted using the different buffers; (**b**) Negative αSyn seeding observed when proteins were extracted from formaldehyde-fixed human brain tissue using buffers 1–6.

Based on previous SAA studies and our observation that paraffin residues contribute to the low protein extraction efficiency, we compared the protein concentrations obtained from the formaldehyde-fixed and FFPE temporal cortex. As expected, BCA measurements showed higher protein concentration from a 4-mm micro-punch of formaldehyde-fixed tissue than from a 4-mm micro-punch of FFPE tissue when extracted with buffers 1, 2, 3, or 6 with xylene-ethanol deparaffinization, and buffers 1, 2, 3, 5, 6 with heptane-methanol deparaffinization (Figure 3a). We evaluated the seeding of αSyn extracted using all six buffers, and although the protein concentration was the highest with formaldehyde-fixed brain tissue, we did not observe seeding (Figure 3b). Because hospitals and brain banks rarely archive formaldehyde-fixed human brain tissue on a large scale for future research, and because our results were negative, we eliminated this tissue type from our study. Further investigation and additional optimization are needed to reverse the crosslinks and extract proteins with seeding potential from 2-week formaldehyde-fixed human brain tissues.

### 3.3. Optimization of FFPE Protein Extraction and αSyn SAA

#### 3.3.1. FFPE Protein Extraction with Heat-Induced Antigen Retrieval and Dissociation

**Figure 4:**
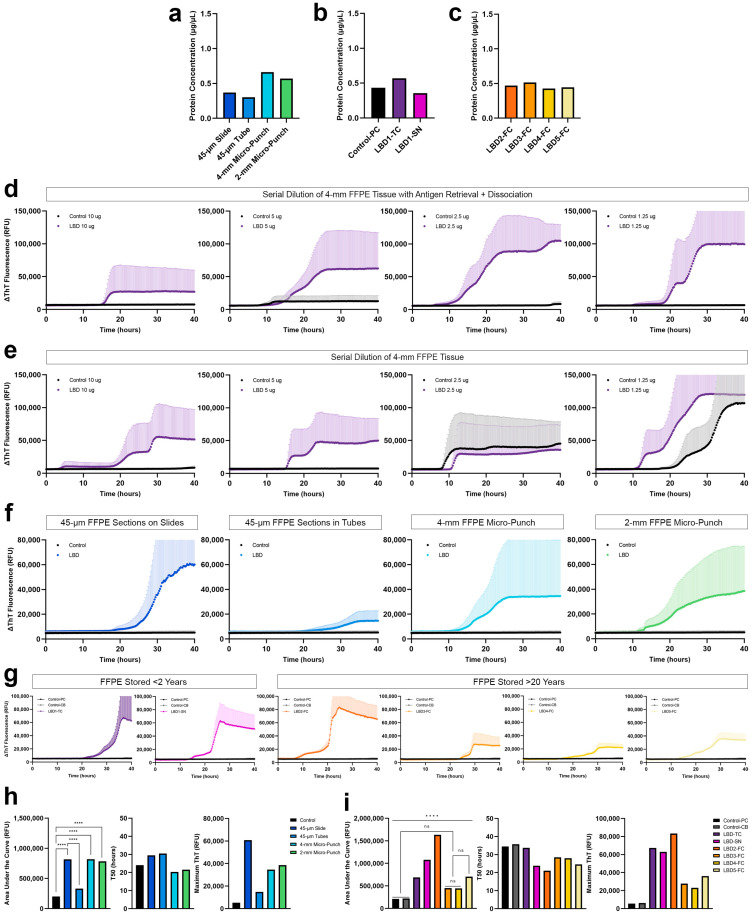
Optimization of αSyn SAA using proteins extracted with antigen retrieval and dissociation. (**a**) Comparison of protein yields obtained with different tissue preparations; (**b**) Protein yields obtained from FFPE parietal cortex of control, and temporal cortex and substantia nigra of LBD subject, that have been archived for <2 years (LBD 1); (**c**) Protein yields obtained from FFPE frontal cortex of four LBD subjects that have been archived for >20 years (LBD 2–5); (**d**) αSyn seeding of serially diluted total protein from PBS-soluble fractions, extracted using antigen retrieval and dissociation prior to protein extraction; (**e**) αSyn seeding of serially diluted total protein from PBS-soluble fractions, extracted without antigen retrieval or dissociation; (**f**) Evaluation of αSyn seeding potential using 5 µg of total protein from PBS-soluble fractions, extracted from different tissue preparations using antigen retrieval and dissociation prior to protein extraction; (**g**) Inter- and intra-subject heterogeneity of αSyn seeding activity in FFPE LBD human brain tissues archived for <2 years (*n* = 1; temporal cortex and substantia nigra) and >20 years (*n* = 4; frontal cortex) with FFPE control human brain tissues (*n* = 1; parietal cortex and cerebellum); (**h**) Area under the curve, T50 and maximum ThT values for different tissue preparations; (**i**) Area under the curve, T50 and maximum ThT values for inter- and intra-subject heterogeneity of αSyn seeding. ****, *p* < 0.0001 by one-way analysis of variance (ANOVA) with Tukey’s multiple comparison test.

Conventional protein extraction from FFPE tissue was inadequate to extract a sufficient amount of protein that can be used to seed αSyn SAA. Thus, based on previous literature emphasizing the importance of heat exposure for crosslink reversal, we incorporated a heat-induced antigen retrieval together with dissociation prior to protein extraction using a commercially available kit and, in addition, resorted to a simple PBS extraction buffer. Using xylene-ethanol deparaffinization and subsequent antigen retrieval and dissociation, we observed a more than two-fold increase in the yield of proteins extracted with buffer 1, even with a 2-mm micro-punch (Figure 4a). Specifically, protein concentrations from highest to lowest were as follows: 4-mm FFPE micro-punch > 2-mm FFPE micro-punch > 45-µm FFPE sections deparaffinized on slide > 45-µm FFPE sections deparaffinized in tubes. Proteins were also extracted from the 2-mm micro-punch of the temporal cortex, and substantia nigra of the test LBD subject archived < 2 years (Figure 4b) and the frontal cortex of four LBD subjects archived for >20 years (Figure 4c) with similar protein yields.

#### 3.3.2. Optimization of αSyn SAA Using Proteins Extracted from FFPE

We compared the αSyn seeding from 4-mm FFPE micro-punch using buffer 1, with and without the additional antigen retrieval and dissociation prior to protein extraction. When we evaluated the seeding of serially diluted proteins extracted with antigen retrieval and dissociation, we observed consistent αSyn seeding across the four LBD replicates with 5 and 10 µg of protein (Figure 4d). We observed lower maximum ThT with increasing total protein amount, which is likely due to the presence of other macromolecules that act as potential inhibitors in more concentrated samples, potentially suppressing the SAA reactions. The control case was negative for all four dilutions. However, with proteins extracted without the antigen retrieval and dissociation, we observed that the seeding of αSyn was inconsistent across the four replicates for all four dilutions, with the control case also showing positive curves with 1.25 and 1.50 µg of protein (Figure 4e). Without the antigen retrieval and dissociation prior to protein extraction, the distinction between LBD and control was unclear, and even a 4-mm micro-punch was not enough to consistently seed misfolded αSyn in FFPE LBD tissue. In contrast, 5 µg of proteins extracted with antigen retrieval and dissociation was sufficient to generate consistent, positive αSyn seeding across the four replicates in FFPE LBD subjects while showing negative curves for controls. Therefore, we used 5 µg of total protein in all of our SAAs.

#### 3.3.3. αSyn Seeding of Proteins Extracted from FFPE Tissue with Different Tissue Preparations

Comparing the seeding of αSyn between different types of tissue preparations, both 4-mm and 2-mm micro-punch of FFPE temporal cortex from LBD subject showed positive curves (Figure 4f) that were significantly different (*p* < 0.0001) from the parietal cortex of control (Figure 4h). Therefore, we determined that a 2-mm micro-punch was sufficient for future experiments. Interestingly, proteins extracted from 45-µm FFPE sections on slides were not only significantly different (*p* < 0.0001) from the control but were also significantly different (*p* < 0.0001) compared to seeding of proteins extracted from 45-µm FFPE sections in tubes (Figure 4h). During deparaffinization, we observed that some areas of the free-floating FFPE scrolls were not entirely exposed to the xylene despite the rigorous vortexing. We suspect that insufficient deparaffinization of FFPE sections in tubes may have contributed to the significantly lower and delayed seeding of αSyn, compared to those deparaffinized on slides. Therefore, FFPE tissues deparaffinized and rehydrated on slides prior to protein extraction, allows thorough deparaffinization, resulting in increased protein amount during extraction and more reliable αSyn seeding activity.

#### 3.3.4. αSyn Seeding of Proteins Extracted from FFPE Tissue Archived for >20 Years

To test whether seeding is preserved in FFPE human brain tissue that was archived for >20 years, we performed SAA in the frontal cortex of four additional LBD subjects archived > 20 years, the temporal cortex and substantia nigra of the test LBD subject archived < 2 years, and the parietal cortex and cerebellum of the test control subject archived < 2 years. Not only did we observe αSyn seeding, but also inter- and intra-subject variability in seeding activity (Figure 4g). αSyn seeding was significantly different (*p* < 0.0001) between the temporal cortex and substantia nigra of LBD 1 that had been archived for <2 years, and αSyn seeding of the LBD 2 frontal cortex was significantly different (*p* < 0.0001) from the frontal cortex of LBD 3–5 (Figure 4i). All LBD curves were significantly different (*p* < 0.0001) from both regions of the control except LBD 3 and LBD 4.

### 3.4. Comparison of Protein Extraction Efficiency and αSyn Seeding Activity with and without Dissociation

**Figure 5:**
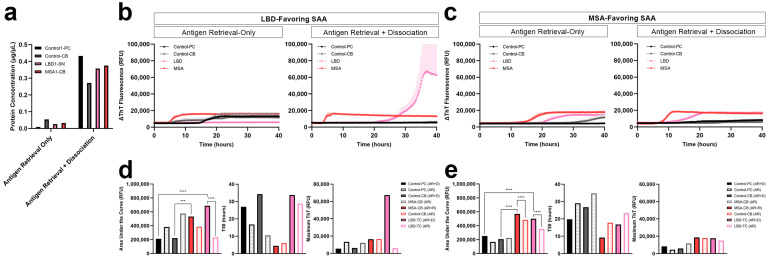
Comparison of αSyn seeding activity between LBD and MSA FFPE brain tissue, extracted with or without dissociation. (**a**) Total protein yield obtained from 2-mm FFPE micro-punch of the parietal cortex and cerebellum of test control subject, temporal cortex of test LBD subject and cerebellum of test MSA subject, extracted with antigen retrieval-only or both antigen retrieval and dissociation prior to protein extraction using PBS; (**b**) Evaluation of disease-specific αSyn seeding in LBD-favouring SAA and comparison of αSyn seeding activity of proteins extracted from dissociated or non-dissociated (antigen retrieval-only) tissues; (**c**) Evaluation of disease-specific αSyn seeding in MSA-favouring SAA and comparison of αSyn seeding of proteins extracted from dissociated or non-dissociated (antigen retrieval-only) tissues; (**d**) Area under the curve, T50 and maximum ThT value for comparison of αSyn seeding activity in PBS-soluble fractions extracted with and without dissociation using the LBD-favouring SAA; (**e**) Area under the curve, T50 and maximum ThT value for comparison of αSyn seeding activity in PBS-soluble fractions extracted with and without dissociation using the MSA-favouring SAA. ***, *p* < 0.001; ****, *p* < 0.0001 by one-way analysis of variance (ANOVA) with Tukey’s multiple comparison test.

Taking into consideration the vigorous tissue treatment in the dissociation step that may lead to loss of proteins during the process, we tested whether the dissociation step could be eliminated. We used a 2-mm FFPE micro-punch of the parietal cortex and cerebellum from the test control subject, the substantia nigra from the test LBD subject, and the cerebellum from the test MSA subject. In order to compare both LBD and MSA, we used both LBD-favouring and MSA-favouring SAA protocols previously established in our group. In all FFPE tissue of control, LBD and MSA subjects, the yield of protein extracted from tissue with only antigen retrieval was significantly lower compared to those with both antigen retrieval and dissociation prior to protein extraction (Figure 5a). Consistent with our extraction results, proteins extracted from LBD and MSA FFPE tissue with only the antigen retrieval showed a longer lag phase and lower AUC compared to those that were extracted after both the antigen retrieval and dissociation. On the other hand, the control FFPE tissue showed spontaneous aggregation when proteins were extracted with antigen retrieval-only.

The LBD-favouring SAA amplifies the seeding of misfolded αSyn in LBD more than MSA. αSyn seeding of LBD was negative when proteins were extracted with antigen retrieval-only, while seeding was observed with both antigen retrieval and dissociation, demonstrating a significant difference (*p* < 0.0001) in seeding between LBD tissues with and without dissociation (Figure 5b,d). On the other hand, the MSA-favouring SAA amplifies the seeding of misfolded αSyn in MSA more than LBD. Using the MSA-favouring protocol, αSyn seeding of LBD had lower AUC when extracted using antigen retrieval-only compared to those extracted with both antigen retrieval and dissociation (Figure 5c). This shows a significant difference (*p* < 0.0001) between αSyn seeding of LBD tissues with and without dissociation (Figure 5e). Lastly, when proteins were extracted with antigen retrieval-only, αSyn seeding of MSA showed lower AUC compared to those extracted with both the antigen retrieval and dissociation, demonstrating a significant difference (*p* < 0.0001) in seeding between MSA tissues with and without dissociation (Figure 5e).

### 3.5. Replication of Disease-Specific Seeding Patterns in FFPE Human Brain Tissue

**Figure 6:**
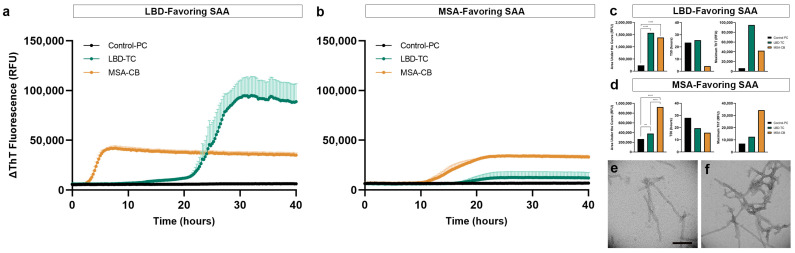
Biochemical and ultra-structural validation of preserved αSyn disease-specificity in LBD and MSA FFPE brain tissue. (**a**) Disease-specific αSyn seeding differences observed in LBD-favouring SAA; (**b**) Disease-specific αSyn seeding differences observed in MSA-favouring SAA; (**c**) Area under the curve, T50 and maximum ThT value for comparison of αSyn seeding activity in LBD, MSA and control FFPE human brain tissue using the LBD-favoring SAA; (**d**) Area under the curve, T50 and maximum ThT value for comparison of αSyn seeding activity in LBD, MSA and control FFPE human brain tissue using the MSA-favoring SAA; (**e**) TEM confirmation of thin, occasional twists observed in SAA-generated αSyn fibrils using LBD FFPE temporal cortex; (**f**) TEM confirmation of thick, intertwined twists observed in SAA-generated αSyn fibrils using MSA FFPE cerebellum. **, *p* < 0.01; ****, *p* < 0.0001 by one-way analysis of variance (ANOVA) with Tukey’s multiple comparison test. Scale bar in (**e**) represents 100 nm.

To further validate our optimized protein extraction and SAA results, we compared the seeding of additional LBD and MSA subjects using 5 µg of extracted proteins from 2-mm FFPE micro-punch, deparaffinized with xylene-ethanol, and processed with antigen retrieval and dissociation prior to protein extraction using buffer 1. With LBD-favouring SAA, LBD showed the highest AUC and maximum ThT value compared to MSA, while MSA showed decreased AUC and lower maximum ThT compared to LBD, amplifying the LBD with higher contrast (Figure 6a). αSyn seeding of LBD was significantly different (*p* < 0.0001) from the control (Figure 6c). With the MSA-favouring SAA, we observed higher AUC in MSA compared to LBD, while misfolded αSyn from LBD showed positive seeding but with lower AUC compared to MSA, amplifying the MSA curve with higher contrast (Figure 6b). αSyn seeding of MSA was significantly different from the control (*p* < 0.0015) and LBD (*p* < 0.0001) (Figure 6d). We did not observe seeding in the control subject. This is consistent with our previous findings in the post-mortem frozen human brain tissue using the LBD- and MSA-favouring SAA protocol [20,21]. Most importantly, αSyn seeding differences between LBD and MSA were transmissible by templating when seeded with SAA end-products in a subsequent LBD-favouring SAA reaction, and both curves were significantly different (*p* < 0.0001) compared to each other (Figure S2).

Altogether, we have confirmed that FFPE human brain tissue preserves seeding potential in short and long-term archived materials and, most importantly, shows disease-specific seeding between LBD and MSA subjects. Our optimized protocol is outlined in Table S1.

### 3.6. Structural Validation of Disease-Specific FFPE-Derived αSyn

To confirm the structural differences between LBD and MSA, as reported in the literature, we evaluated the fibrils generated during the seeding assays under TEM. We used our optimized protein extraction protocol to extract protein from an additional FFPE LBD and MSA subject (LBD 6 and MSA 2), archived for <2 years. We confirmed the disease-specific, ultra-structural differences between the αSyn fibrils in LBD and MSA, where fibrils generated from LBD-seeded reaction had long and thin fibrils with occasional twists (Figure 6e) compared to those generated from MSA-seeded reaction that had thinner fibrils with intertwined and frequently twisted strands (Figure 6f).

## 4. Discussion

In this study, we have demonstrated that disease-specific seeding of misfolded αSyn, including ultra-structural differences of the generated fibrils, is preserved in FFPE human brain tissue (Figure 5). Extraction of a sufficient amount of high-quality proteins from FFPE tissue has consistently been reported to be challenging [28,29]. Many studies have investigated different deparaffinization methods and extraction buffers, including commercially available FFPE protein extraction kits, to achieve a higher protein yield from FFPE tissue [47,48,49,50]. Although commercially available protein extraction kits can be effective for proteomic analysis, the extraction leaves the proteins suspended in extraction buffers that are composed of proprietary reagents, which can alter the performance of SAAs. To overcome these limitations, our study has established an optimized protein extraction protocol that uses antigen retrieval and dissociation to remove paraffin residues and reverse crosslinks, leaving a pellet of dissociated tissue that can be used for protein extraction with a simple PBS buffer commonly used to homogenize post-mortem frozen brain tissue for SAAs. Our protocol also circumvents buffer carry-over into SAAs, unlike FFPE extraction kits and other extraction methods, preserving the original SAA buffer composition. Furthermore, the tissue pellet can be resuspended in any buffer of choice, providing wide applicability to other SAA protocols or biochemical analyses.

Previous SAA studies have reported the detection of misfolded αSyn in formaldehyde-fixed human tissue of PD, incidental LBD and MSA subjects and more recently, Hepker et al. developed a kinetic assay seeding ability recovery (KASAR) protocol using FFPE core of globus pallidus with great sensitivity and specificity [51,52,53]. However, despite the successful findings, brain tissues were fixed for a relatively short amount of time (18 h) or in a less concentrated formaldehyde solution (4%) or fixed in paraformaldehyde that does not penetrate the tissue as rapidly. Unfortunately, this does not reflect the tissue processing procedures used in most hospitals and brain banks. In this study, all our brain tissue has been processed in 10% neutral buffered formaldehyde for 2 to 3 weeks prior to systemic tissue processing and paraffin-embedding, highlighting the applicability of our findings to archived FFPE human brain tissues across many hospitals, brain banks and research laboratories. In addition, we have obtained high protein yield and observed αSyn seeding in FFPE human brain tissues that were fixed and embedded in paraffin more than 20 years ago. Most importantly, to preserve the disease-specific αSyn seeding, we avoided long exposure to high temperatures for crosslink reversal, as it is unclear whether prolonged exposure to heat alters the original fibril structure or results in a partial loss of proteins.

Furthermore, with our streamlined protocol, including antigen retrieval and dissociation, the proteins extracted from a 2-mm FFPE micro-punch were sufficient to generate positive seeding activity. We have also demonstrated that disease-specific seeding activity is preserved in FFPE brain tissue. We found that MSA subjects consistently showed higher seeding activity, with shorter lag phases compared to LBD subjects in both conditions that favour either LBD or MSA seeding. The optimized method also offers to capture the ultra-structural differences between LBD and MSA fibrils when αSyn fibrils generated from our FFPE SAA are evaluated under TEM. This finding is consistent with the structural variability reported in the literature, where thin αSyn fibril formation in LBD subjects may be due to the single protofilament structure observed with cryo-electron microscopy and thick intertwined fibril formation in MSA subjects can be due to fibril structure made of two different protofilaments [10,11].

A variety of αSyn polymorphs may exist in different subcellular locations in a single human brain tissue. It is unknown whether a subset of proteins (i.e., membrane or cytoplasmic) are preferentially extracted during the FFPE protein extraction process or whether certain processes in the protein extraction step induce a partial removal of either soluble or insoluble proteins, emphasizing the need for cautious interpretation. Despite this limitation, we observed clear differences between LBD and MSA subjects using our optimized FFPE protein extraction and SAA method. With frozen tissue remaining as the gold standard for protein analysis and seeding assays, our streamlined protein extraction protocol can reveal the disease-specific seeding activity preserved in FFPE human brain tissue. This will allow the re-evaluation of large collections of archived FFPE tissues, paving the path for future protein-based studies.

## 5. Conclusions

To the best of our knowledge, this study is the first to recapitulate disease-specific differences between LBD and MSA in post-mortem FFPE human brain tissue. We have developed a streamlined protein extraction protocol that extends the utility of FFPE tissue beyond immunohistochemical purposes. Furthermore, our study allows the expansion of research on distinct αSyn polymorphs and further subclassification of disease based on seeding differences, with the use of largely available and highly characterized FFPE human brain tissue as an alternative to frozen tissue.

## Figures and Tables

**Table 1:** Demographics and neuropathological diagnosis.

Case	Sex	Age at Death	PMD (Hours)	Storage (Years)	ABC Score *	CAA	Lewy Body Pathology	Lewy Body Braak Stage	LATE-NC Stage	Other Neuropathological Diagnosis
Control 1	F	52	28	3	None	None	None	None	None	Early Fahr Disease
LBD 1	M	74	NA	2	A1B1C1	Type 2	Neocortical	Stage 5	None	None
LBD 2	F	73	NA	26	A2B3C3	None	Neocortical	Stage 5	Stage 2	None
LBD 3	F	73	2.5	25	A2B3C2	None	Limbic	Stage 4	Stage 2	None
LBD 4	F	82	24–36	25	A2B2C2	None	Neocortical	Stage 5	None	ARTAG Medial Temporal GM, WM, Brainstem
LBD 5	F	62	15	25	A3B3C3	None	Neocortical	Stage 5	Stage 1	None
LBD 6	M	78	40	3	A3B2C1	Type 2	Neocortical	Stage 5	Stage 2	Meningioma Right Frontal
MSA 1	F	68	4	36	A1B1C0	Type 2	None	None	None	ARTAG Medial Temporal WM
MSA 2	M	64	NA	3	A0B1C0	None	None	None	None	PART (Braak stage II), AGD (Stage II)

* Subjects were given an ABC score based on the neuropathologic assessment of Alzheimer’s disease in ref [43]. Abbreviations: PMD, post-mortem delay; CAA, cerebral amyloid angiopathy; LATE-NC, limbic-predominant age-related TDP-43 encephalopathy-neuropathological change; LBD, Lewy body disease; Aβ, amyloid-β; ARTAG, age-related tau astrogliopathy; GM, grey matter; WM, white matter; MSA, multiple system atrophy; PART, primary age-related tauopathy; AGD, argyrophilic grain disease; NA, not available.

**Table 2:** Protein extraction buffer compositions.

Buffer	Buffer Composition
Buffer 1	1 × Phosphate-Buffered Saline (pH 7.4) Protease inhibitor
Buffer 2	1 × Phosphate-Buffered Saline (pH 7.4) 0.01% β-mercaptoethanol Protease inhibitor
Buffer 3	1 × Tris Buffered Saline (50 mM Tris-HCl (pH 7.5) 150 mM NaCl) Protease inhibitor
Buffer 4	50 mM Tris-HCl (pH 8.0) Protease inhibitor
Buffer 5	50 mM Tris-HCl (pH 8.0) 1% Nonyl Phenoxypolyethoxylethanol-40 150 mM NaCl Protease inhibitor
Buffer 6	50 mM Tris-HCl (pH 8.0) 1% Nonyl Phenoxypolyethoxylethanol-40 Protease inhibitor

## Data Availability

The data presented in this study are available on request from the corresponding author.

## Supplementary Materials

The following supporting information can be downloaded at: https://www.mdpi.com/article/10.3390/biom13060936/s1, Figure S1: Immunohistochemistry of disease-specific αSyn detected in FFPE subjects; Figure S2: αSyn seeding differences between FFPE LBD and MSA are transmissible by templating using SAA end-products; Table S1: Summary of optimized protein extraction protocol for FFPE brain tissue.

## References

[1] Maroteaux L., Campanelli J., Scheller R. (1988). Synuclein: A Neuron-Specific Protein Localized to the Nucleus and Presynaptic Nerve Terminal. J. Neurosci..

[2] Fujiwara H., Hasegawa M., Dohmae N., Kawashima A., Masliah E., Goldberg M.S., Shen J., Takio K., Iwatsubo T. (2002). α-Synuclein Is Phosphorylated in Synucleinopathy Lesions. Nat. Cell Biol..

[3] Vilar M., Chou H.T., Lührs T., Maji S.K., Riek-Loher D., Verel R., Manning G., Stahlberg H., Riek R. (2008). The Fold of α-Synuclein Fibrils. Proc. Natl. Acad. Sci. USA.

[4] Kovacs G.G. (2019). Molecular Pathology of Neurodegenerative Diseases: Principles and Practice. J. Clin. Pathol..

[5] Spillantini M.G., Schmidt M.L., Lee V.M.-Y., Trojanowski J.Q., Jakes R., Goedert M. (1997). α-Synuclein in Lewy Bodies. Nature.

[6] Papp M.I., Kahn J.E., Lantos P.L. (1989). Glial Cytoplasmic Inclusions in the CNS of Patients with Multiple System Atrophy (Striatonigral Degeneration, Olivopontocerebellar Atrophy and Shy-Drager Syndrome). J. Neurol. Sci..

[7] Gilman S., Wenning G.K., Low P.A., Brooks D.J., Mathias C.J., Trojanowski J.Q., Wood N.W., Colosimo C., Durr A., Fowler C.J. (2008). Second Consensus Statement on the Diagnosis of Multiple System Atrophy. Neurology.

[8] Bousset L., Pieri L., Ruiz-Arlandis G., Gath J., Jensen P.H., Habenstein B., Madiona K., Olieric V., Böckmann A., Meier B.H. (2013). Structural and Functional Characterization of Two Alpha-Synuclein Strains. Nat. Commun..

[9] Van der Perren A., Gelders G., Fenyi A., Bousset L., Brito F., Peelaerts W., Van den Haute C., Gentleman S., Melki R., Baekelandt V. (2020). The Structural Differences between Patient-Derived α-Synuclein Strains Dictate Characteristics of Parkinson’s Disease, Multiple System Atrophy and Dementia with Lewy Bodies. Acta Neuropathol..

[10] Schweighauser M., Shi Y., Tarutani A., Kametani F., Murzin A.G., Ghetti B., Matsubara T., Tomita T., Ando T., Hasegawa K. (2020). Structures of α-Synuclein Filaments from Multiple System Atrophy. Nature.

[11] Yang Y., Shi Y., Schweighauser M., Zhang X., Kotecha A., Murzin A.G., Garringer H.J., Cullinane P.W., Saito Y., Foroud T. (2022). Structures of α-Synuclein Filaments from Human Brains with Lewy Pathology. Nature.

[12] Lau A., So R.W.L., Lau H.H.C., Sang J.C., Ruiz-Riquelme A., Fleck S.C., Stuart E., Menon S., Visanji N.P., Meisl G. (2020). α-Synuclein Strains Target Distinct Brain Regions and Cell Types. Nat. Neurosci..

[13] Telling G.C., Parchi P., DeArmond S.J., Cortelli P., Montagna P., Gabizon R., Mastrianni J., Lugaresi E., Gambetti P., Prusiner S.B. (1996). Evidence for the Conformation of the Pathologic Isoform of the Prion Protein Enciphering and Propagating Prion Diversity. Science.

[14] Groveman B.R., Orrù C.D., Hughson A.G., Raymond L.D., Zanusso G., Ghetti B., Campbell K.J., Safar J., Galasko D., Caughey B. (2018). Rapid and Ultra-Sensitive Quantitation of Disease-Associated α-Synuclein Seeds in Brain and Cerebrospinal Fluid by ASyn RT-QuIC. Acta Neuropathol. Commun..

[15] Wang Z., Becker K., Donadio V., Siedlak S., Yuan J., Rezaee M., Incensi A., Kuzkina A., Orrú C.D., Tatsuoka C. (2021). Skin α-Synuclein Aggregation Seeding Activity as a Novel Biomarker for Parkinson Disease. JAMA Neurol..

[16] Biancalana M., Koide S. (2010). Molecular Mechanism of Thioflavin-T Binding to Amyloid Fibrils. Biochim. Biophys. Acta-Proteins Proteom..

[17] Buell A.K., Galvagnion C., Gaspar R., Sparr E., Vendruscolo M., Knowles T.P.J., Linse S., Dobson C.M. (2014). Solution Conditions Determine the Relative Importance of Nucleation and Growth Processes in α-Synuclein Aggregation. Proc. Natl. Acad. Sci. USA.

[18] Ramis R., Ortega-Castro J., Vilanova B., Adrover M., Frau J. (2020). Unraveling the NaCl Concentration Effect on the First Stages of α-Synuclein Aggregation. Biomacromolecules.

[19] Metrick M.A., do Carmo Ferreira N., Saijo E., Hughson A.G., Kraus A., Orrú C., Miller M.W., Zanusso G., Ghetti B., Vendruscolo M. (2019). Million-Fold Sensitivity Enhancement in Proteopathic Seed Amplification Assays for Biospecimens by Hofmeister Ion Comparisons. Proc. Natl. Acad. Sci. USA.

[20] Martinez-Valbuena I., Visanji N.P., Kim A., Lau H.H.C., So R.W.L., Alshimemeri S., Gao A., Seidman M.A., Luquin M.R., Watts J.C. (2022). Alpha-Synuclein Seeding Shows a Wide Heterogeneity in Multiple System Atrophy. Transl. Neurodegener..

[21] Martinez-Valbuena I., Swinkin E., Santamaria E., Fernandez-Irigoyen J., Sackmann V., Kim A., Li J., Gonzalez-Latapi P., Kuhlman G., Bhowmick S.S. (2022). α-Synuclein Molecular Behavior and Nigral Proteomic Profiling Distinguish Subtypes of Lewy Body Disorders. Acta Neuropathol..

[22] Sutherland B.W., Toews J., Kast J. (2008). Utility of Formaldehyde Cross-Linking and Mass Spectrometry in the Study of Protein–Protein Interactions. J. Mass Spectrom..

[23] Loiacono C.M., Beckwith N., Kunkle R.A., Orcutt D., Hall S.M. (2010). Detection of PrP Sc in Formalin-Fixed, Paraffin-Embedded Tissue by Western Blot Differentiates Classical Scrapie, Nor98 Scrapie, and Bovine Spongiform Encephalopathy. J. Vet. Diagn. Investig..

[24] Priola S.A., Ward A.E., McCall S.A., Trifilo M., Choi Y.P., Solforosi L., Williamson R.A., Cruite J.T., Oldstone M.B.A. (2013). Lack of Prion Infectivity in Fixed Heart Tissue from Patients with Creutzfeldt-Jakob Disease or Amyloid Heart Disease. J. Virol..

[25] Hoover C.E., Davenport K.A., Henderson D.M., Pulscher L.A., Mathiason C.K., Zabel M.D., Hoover E.A. (2016). Detection and Quantification of CWD Prions in Fixed Paraffin Embedded Tissues by Real-Time Quaking-Induced Conversion. Sci. Rep..

[26] Dong T.-T.-T., Akagi A., Nonaka T., Nakagaki T., Mihara B., Takao M., Iwasaki Y., Nishida N., Satoh K. (2021). Formalin RT-QuIC Assay Detects Prion-Seeding Activity in Formalin-Fixed Brain Samples from Sporadic Creutzfeldt–Jakob Disease Patients. Neurobiol. Dis..

[27] Watts J.C. (2019). Calling α-Synuclein a Prion Is Scientifically Justifiable. Acta Neuropathol..

[28] Manne S., Kondru N., Jin H., Serrano G.E., Anantharam V., Kanthasamy A., Adler C.H., Beach T.G., Kanthasamy A.G. (2020). Blinded RT-QuIC Analysis of α-Synuclein Biomarker in Skin Tissue from Parkinson’s Disease Patients. Mov. Disord..

[29] Manne S., Kondru N., Jin H., Anantharam V., Huang X., Kanthasamy A., Kanthasamy A.G. (2020). A-Synuclein Real-Time Quaking-Induced Conversion in the Submandibular Glands of Parkinson’s Disease Patients. Mov. Disord..

[30] Shin C., Han J.Y., Kim S.I., Park S.H., Yang H.K., Lee H.J., Kong S.H., Suh Y.S., Kim H.J., Choi Y.P. (2022). In Vivo and Autopsy Validation of Alpha-Synuclein Seeding Activity Using RT-QuIC Assay in the Gastrointestinal Tract of Patients with Parkinson’s Disease. Park. Relat. Disord..

[31] Fowler C.B., O’Leary T.J., Mason J.T. (2013). Toward Improving the Proteomic Analysis of Formalin-Fixed, Paraffin-Embedded Tissue. Expert Rev. Proteom..

[32] Hoffman E.A., Frey B.L., Smith L.M., Auble D.T. (2015). Formaldehyde Crosslinking: A Tool for the Study of Chromatin Complexes. J. Biol. Chem..

[33] Kamps J.J.A.G., Hopkinson R.J., Schofield C.J., Claridge T.D.W. (2019). How Formaldehyde Reacts with Amino Acids. Commun. Chem..

[34] Giusti L., Lucacchini A. (2013). Proteomic Studies of Formalin-Fixed Paraffin-Embedded Tissues. Expert Rev. Proteom..

[35] Kawashima Y., Kodera Y., Singh A., Matsumoto M., Matsumoto H. (2014). Efficient Extraction of Proteins from Formalin-Fixed Paraffin-Embedded Tissues Requires Higher Concentration of Tris(Hydroxymethyl)Aminomethane. Clin. Proteom..

[36] Shen K., Sun J., Cao X., Zhou D., Li J. (2015). Comparison of Different Buffers for Protein Extraction from Formalin-Fixed and Paraffin-Embedded Tissue Specimens. PLoS ONE.

[37] O’Rourke M.B., Padula M.P. (2016). Analysis of Formalin-Fixed, Paraffin-Embedded (FFPE) Tissue via Proteomic Techniques and Misconceptions of Antigen Retrieval. Biotechniques.

[38] Thacker J.S., Andersen D., Liang S., Zieniewicz N., Trivino-Paredes J.S., Nahirney P.C., Christie B.R. (2021). Unlocking the Brain: A New Method for Western Blot Protein Detection from Fixed Brain Tissue. J. Neurosci. Methods.

[39] Addis M.F., Tanca A., Pagnozzi D., Crobu S., Fanciulli G., Cossu-Rocca P., Uzzau S. (2009). Generation of High-Quality Protein Extracts from Formalin-Fixed, Paraffin-Embedded Tissues. Proteomics.

[40] Rodríguez-Rigueiro T., Valladares-Ayerbes M., Haz-Conde M., Blanco M., Aparicio G., Fernández-Puente P., Blanco F.J., Lorenzo M.J., Aparicio L.A., Figueroa A. (2011). A Novel Procedure for Protein Extraction from Formalin-Fixed Paraffin-Embedded Tissues. Proteomics.

[41] Araújo J.E., Oliveira E., Otero-Glez A., Santos Nores J., Igrejas G., Lodeiro C., Capelo J.L., Santos H.M. (2014). A Comprehensive Factorial Design Study of Variables Affecting Protein Extraction from Formalin-Fixed Kidney Tissue Samples. Talanta.

[42] Dressler F.F., Schoenfeld J., Revyakina O., Vogele D., Kiefer S., Kirfel J., Gemoll T., Perner S. (2022). Systematic Evaluation and Optimization of Protein Extraction Parameters in Diagnostic FFPE Specimens. Clin. Proteom..

[43] Montine T.J., Phelps C.H., Beach T.G., Bigio E.H., Cairns N.J., Dickson D.W., Duyckaerts C., Frosch M.P., Masliah E., Mirra S.S. (2012). National Institute on Aging–Alzheimer’s Association Guidelines for the Neuropathologic Assessment of Alzheimer’s Disease: A Practical Approach. Acta Neuropathol..

[44] Kovacs G.G., Wagner U., Dumont B., Pikkarainen M., Osman A.A., Streichenberger N., Leisser I., Verchère J., Baron T., Alafuzoff I. (2012). An Antibody with High Reactivity for Disease-Associated α-Synuclein Reveals Extensive Brain Pathology. Acta Neuropathol..

[45] Koo H.-J., Lee H.-J., Im H. (2008). Sequence Determinants Regulating Fibrillation of Human α-Synuclein. Biochem. Biophys. Res. Commun..

[46] Mansour A.G., Abou-Khalil P., Bejjani N., Chatila R., Dagher-Hamalian C., Faour W.H. (2017). An Optimized Xylene-Free Protein Extraction Method Adapted to Formalin-Fixed Paraffin Embedded Tissue Sections for Western Blot Analysis. Histol. Histopathol..

[47] Wolff C., Schott C., Porschewski P., Reischauer B., Becker K.-F. (2011). Successful Protein Extraction from Over-Fixed and Long-Term Stored Formalin-Fixed Tissues. PLoS ONE.

[48] Geoui T., Urlaub H., Plessmann U., Porschewski P. (2010). Extraction of Proteins from Formalin-Fixed, Paraffin-Embedded Tissue Using the Qproteome Extraction Technique and Preparation of Tryptic Peptides for Liquid Chromatography/Mass Spectrometry Analysis. Current Protocols in Molecular Biology.

[49] Mansour A., Chatila R., Bejjani N., Dagher C., Faour W.H. (2014). A Novel Xylene-Free Deparaffinization Method for the Extraction of Proteins from Human Derived Formalin-Fixed Paraffin Embedded (FFPE) Archival Tissue Blocks. MethodsX.

[50] García-Vence M., Chantada-Vazquez M.d.P., Sosa-Fajardo A., Agra R., Barcia de la Iglesia A., Otero-Glez A., García-González M., Cameselle-Teijeiro J.M., Nuñez C., Bravo J.J. (2021). Protein Extraction from FFPE Kidney Tissue Samples: A Review of the Literature and Characterization of Techniques. Front. Med..

[51] Becker K., Wang X., Vander Stel K., Chu Y., Kordower J., Ma J. (2018). Detecting Alpha Synuclein Seeding Activity in Formaldehyde-Fixed MSA Patient Tissue by PMCA. Mol. Neurobiol..

[52] Fenyi A., Duyckaerts C., Bousset L., Braak H., Del Tredici K., Melki R. (2021). Seeding Propensity and Characteristics of Pathogenic ASyn Assemblies in Formalin-Fixed Human Tissue from the Enteric Nervous System, Olfactory Bulb, and Brainstem in Cases Staged for Parkinson’s Disease. Cells.

[53] Hepker M., Clabaugh G., Jin H., Kanthasamy A.G. (2023). New Protocol for Kinetic Assay Seeding Ability Recovery “KASAR” from Formalin-Fixed Paraffin-Embedded Tissues. Front. Mol. Biosci..

