# Supplementary material for: Disease-Specific α-Synuclein Seeding in Lewy Body Disease and Multiple System Atrophy Are Preserved in Formaldehyde-Fixed Paraffin-Embedded Human Brain
Source: Biomolecules. 2023 Jun 2;13(6):936. doi: 10.3390/biom13060936 (PMC10296376; doi:10.3390/biom13060936)
Supplement: Supplementary file 1 [file biomolecules-13-00936-s001.zip › biomolecules-2334023-supplementary.docx]

**
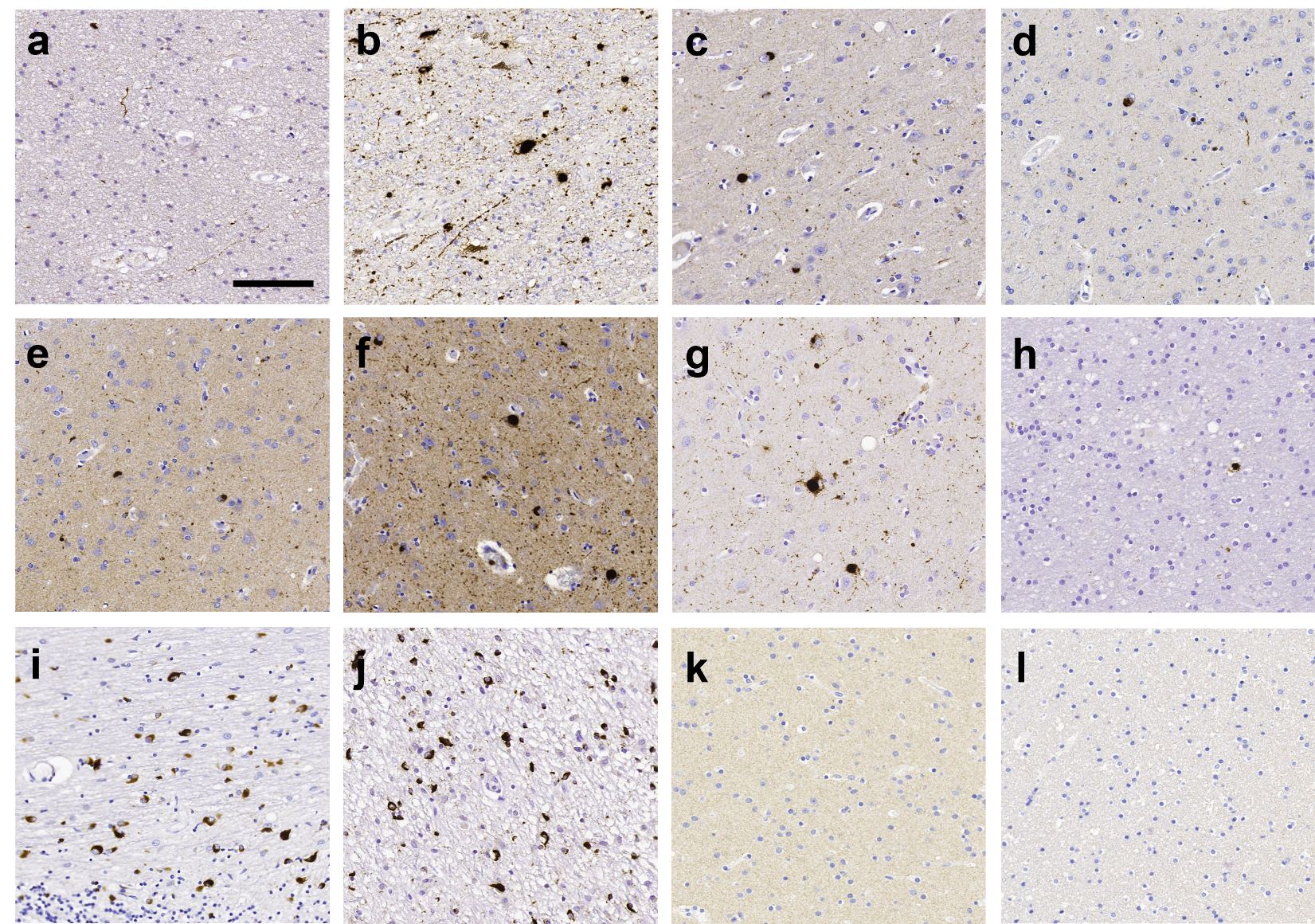
**

**Figure S1.** Immunohistochemistry of disease-specific αSyn detected in FFPE subjects. (**a**) Temporal cortex of LBD 1; (**b**) Substantia nigra of LBD 1; (**c**) Frontal cortex of LBD 2; (**d**) Frontal cortex of LBD 3; (**e**) Frontal cortex of LBD 4; (**f**) Frontal cortex of LBD 5; (**g**) Temporal cortex of LBD 6; (**h**) Temporal white matter of LBD 6; (**i**) Cerebellum of MSA 1; (**j**) Cerebellum of MSA 2; (**k**) Parietal cortex of control 1; (**l**) Parietal white matter of control 1. Scale bar represents 50 μm.


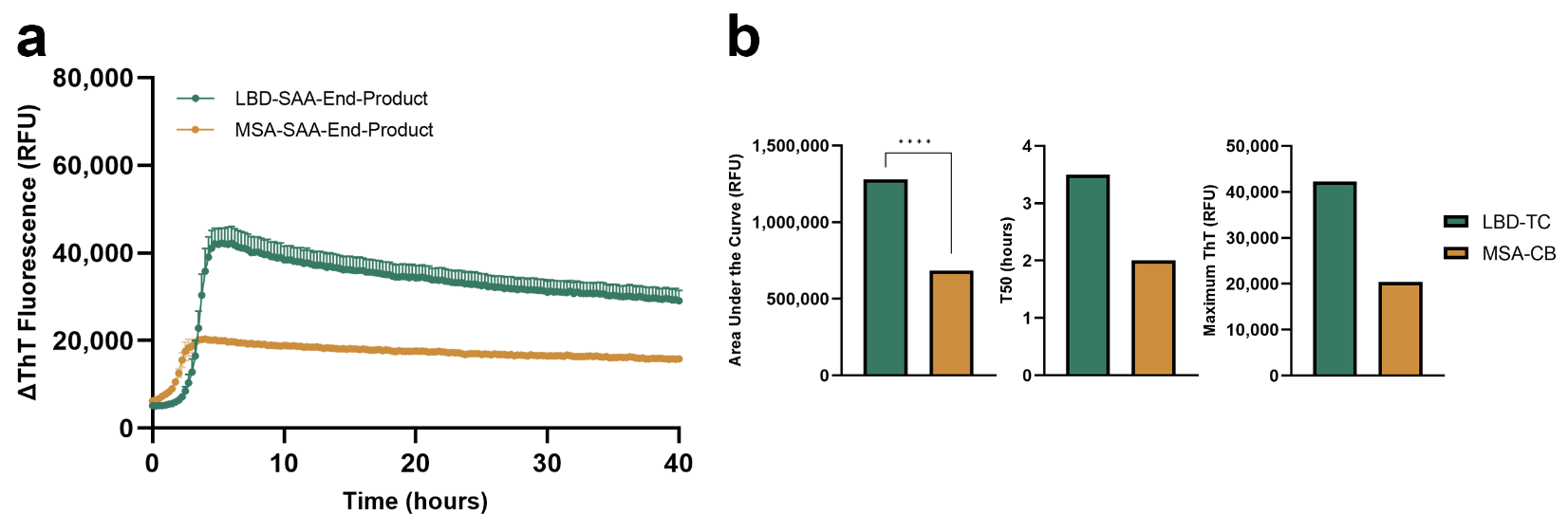


**Figure S2.** αSyn seeding differences between FFPE LBD and MSA are transmissible by templating using SAA end-products. (**a**) Seeding of LBD and MSA SAA end-products in subsequent SAA reaction using LBD-favoring SAA shows transmissibility of αSyn seeding differences; (**b**) Area under the curve, T50 and maximum ThT values for LBD and MSA SAA end-products. ****, p < 0.0001 by unpaired two-tailed parametric t-test.

**Table S1.** Summary of optimized protein extraction protocol for FFPE brain tissue

| **Type** | **Step** | **Protocol** |
| --- | --- | --- |
| **Tissue Collection:**  45-µm Sections on Slide | 1. | Using a microtome, cut 10 sections of the pre-chilled and trimmed FFPE block at 4.5-µm-thickness and float the paraffin ribbon on 40°C water bath with ultra-pure H_2_O. |
|  | 2. | Using a tweezer to separate the ribbons, pick out the floating FFPE sections with a positively charged microscope slide and dry in a slide rack, overnight at room temperature. |
| **Tissue Collection:**  2-mm Micro-Punch | 1. | Place the FFPE block with the corresponding base mold into the paraffin-melting chamber in the embedding machine, or into a 62°C oven for 10 minutes to melt the wax. |
|  | 2. | Using a disposable biopsy punch with or without plunger, carefully make a micro-puncture of the desired region on the FFPE block and lift the biopsy punch in a slow twisting motion. Release the sample into the C-tube. |
| **Deparaffinization** | 1. | 2X 10 minutes fresh xylene, 2X 5 minutes fresh 100% ethanol, 5 minutes 95% ethanol, 5 minutes 80% ethanol, 5 minutes 70% ethanol, 5 minutes 50% ethanol, 5 minutes ultra-pure H_2_O.  *For sections in slide racks, use a staining dish containing xylene and ethanol. For micro-punches, add 1-2 mL of the deparaffinization and rehydration solutions into the C-tube using a micro-pipette.* |
|  | 2. | For sections, use a microtome blade coated in Sigmacote (low protein-binding) to carefully scrape the sections and place in the C-tubes. |
| **Antigen Retrieval & Dissociation** | 1. | Follow manufacturer’s instructions with slight modification: |
|  |  | After completing the dissociation step, briefly centrifuge the C-tubes and do not use a filter to discard the dissociated tissue. Instead, add 1 mL of ice-cold 1X PBS spiked with protease inhibitor, transfer to 1.5 mL low protein binding tubes and centrifuge for 5 minutes, 4°C at 5000 *g*. Carefully discard the supernatant and resuspend in 1 mL ice-cold 1X PBS spiked with protease inhibitor. Centrifuge for 5 minutes, 4°C at 5000 *g* and discard the supernatant. |
| **Protein Extraction** | 1. | Tare the scale after measuring an empty 1.5 mL low protein binding tube. Weigh the tissue pellet and record the measurement. |
|  | 2. | Add 10% w/v ice-cold 1X PBS spiked with protease inhibitor into the low protein-binding tubes and re-suspend by pipetting up and down. Transfer the re-suspended pellets into the low protein-binding tissue homogenizing tubes and homogenize using Beadbeater at high speed for 30 seconds. Place tubes on ice and repeat 2 additional times, at 5-minute intervals. |
|  | 3. | Transfer the homogenized mixture into 1.5 mL low protein-binding Eppendorf tubes using low protein-binding micro-pipette tips and place on ice for 10 minutes followed by vortexing. Repeat 3 additional times. |
|  | 4. | Centrifuge the tubes for 10 minutes, 4°C at 10000 *g.* Transfer the supernatant into a clean 1.5 mL low protein binding tube, mix well and aliquot into 0.5 mL low protein binding tubes as necessary. Perform BCA to quantify the proteins in each sample. |
